# Overexpression of catalase in mitochondria mitigates changes in hippocampal cytokine expression following simulated microgravity and isolation

**DOI:** 10.1038/s41526-021-00152-w

**Published:** 2021-07-06

**Authors:** Linda Rubinstein, Ann-Sofie Schreurs, Samantha M. Torres, Sonette Steczina, Moniece G. Lowe, Frederico Kiffer, Antiño R. Allen, April E. Ronca, Marianne B. Sowa, Ruth K. Globus, Candice G. T. Tahimic

**Affiliations:** 1 Universities Space Research Association, Columbia, MD USA; 2 Space Biosciences Division, NASA Ames Research Center, Moffett Field, CA USA; 3 KBR, Houston, TX USA; 4 Blue Marble Space Institute of Science, Seattle, WA 98154 USA; 5 Division of Radiation Health, University of Arkansas for Medical Sciences, Little Rock, AR USA; 6 Department of Pharmaceutical Sciences, University of Arkansas for Medical Sciences, Little Rock, AR USA; 7 Neurobiology and Developmental Sciences, University of Arkansas for Medical Sciences, Little Rock, AR USA; 8 Wake Forest Medical School, Winston-Salem, NC USA; 9 Department of Biology, University of North Florida, Jacksonville, FL USA

**Keywords:** Interleukins, Physiology

## Abstract

Isolation on Earth can alter physiology and signaling of organs systems, including the central nervous system. Although not in complete solitude, astronauts operate in an isolated environment during spaceflight. In this study, we determined the effects of isolation and simulated microgravity solely or combined, on the inflammatory cytokine milieu of the hippocampus. Adult female wild-type mice underwent simulated microgravity by hindlimb unloading for 30 days in single or social (paired) housing. In hippocampus, simulated microgravity and isolation each regulate a discrete repertoire of cytokines associated with inflammation. Their combined effects are not additive. A model for mitochondrial reactive oxygen species (ROS) quenching via targeted overexpression of the human catalase gene to the mitochondria (MCAT mice), are protected from isolation- and/or simulated microgravity-induced changes in cytokine expression. These findings suggest a key role for mitochondrial ROS signaling in neuroinflammatory responses to spaceflight and prolonged bedrest, isolation, and confinement on Earth.

## Introduction

Isolation is an important feature of the spaceflight environment. Interplanetary missions (e.g., trip to Mars) will involve mission crew operating in isolated environments for prolonged periods of time. On Earth, isolation has profound effects on the central nervous system (CNS) including behavioral changes, upregulated oxidative stress pathways, neuroinflammatory responses, as well as brain cytokine alterations^1–3^. In addition, isolation worsens aging-related cognitive impairments and immune deficits^4^.

Spaceflight also can lead to immune dysfunction as well as reactivation of latent viruses^5–8^. International Space Station (ISS) crew members on a 6-month spaceflight mission had diminished T/NK cell function^9^ and increased inflammatory plasma cytokine levels^10^. In shorter duration missions (9–14 days), changes in the distribution and function of peripheral leukocytes were also detected in astronauts^11^. Further, the subset of astronauts who experienced viral shedding also displayed increased plasma cytokine protein levels in their plasma during flight^12^, linking dysregulated cytokine levels with impaired immune function.

We showed previously that isolation in combination with simulated microgravity by hindlimb unloading (HU) led to a reduction in the percentage of circulating CD4+ immune cells, which was not observed in social housed HU animals^13^. This finding suggests that isolation can modify immune responses to simulated spaceflight and that the combination of these two spaceflight stressors can lead to discrete immune deficits. However, the combined effects of isolation and microgravity on neuroimmune outcomes are not defined. Excess production of free radicals (e.g., reactive oxygen species, ROS) have been linked to neuroinflammation^14^. Yet there have been few studies exploring oxidative damage as a mechanism by which space environmental factors, singly or in combination, mediate aspects of CNS immune responses. To address these gaps in knowledge, HU was conducted on paired (social) and single housed wild-type and MCAT mice, a genetic model for mitochondrial ROS quenching. MCAT mice overexpress the human catalase gene targeted to the mitochondria^15^, a major site of cellular ROS production. MCAT mice display decreased oxidative damage and H_2_O_2_ production.

The MCAT transgenic model^15^ was developed to enable testing various aspects of the free radical theory of aging^16^, which posits that cells, tissues, and organisms age due to an accumulation of oxidative damage via excess production of ROS. Overexpression of catalase when targeted specifically to the mitochondria, but not when targeted to the nucleus or cytoplasm, prolongs murine lifespan and reduces age-related cardiovascular^17–19^ and cognitive deficits^20^ sharing features with known or anticipated effects of spaceflight^21–25^. Findings from human astronauts long ago led to the proposal that spaceflight and advancing age share physiological consequences^26^, leading us to question if they also share molecular mechanisms. Therefore, we determined whether the MCAT transgene also is protective using a ground-based model that simulates important aspects of spaceflight, microgravity, and isolation.

MCAT mice show increased median and maximum lifespans (5 months, and 5.5 months, respectively)^15^ and are protected from a number of aging-associated pathologies. For example, MCAT mice display protection from cardiovascular deficits^17–19^ and Alzheimer’s disease-related amyloid deposition^27^. Further, the brains of MCAT mice indicate the rescue of simulated space radiation-induced decrements in neurogenesis^28^ and neuronal spine morphology^29^. The overexpression of catalase also quenches mitochondrial oxidative stress in macrophages^30^, suggesting that catalase can impact oxidative signaling in inflammatory cells. MCAT mice also exhibit enhanced hippocampal spatial learning, memory, and reduced contextual fear conditioning^15,20^.

Altered cognitive function is thought to be one of the risks associated with long-duration spaceflight^31^. The production of ROS, pro-inflammatory cytokines, chemokines, and neurotrophic factors are involved in hippocampal neuropathy. Prolonged exposure to simulated microgravity by HU is associated with increased hippocampal oxidative stress biomarkers, which may increase injury^32^. Proteomic analysis of the hippocampus in HU mice reveals changes in structural proteins coupled with the loss of proteins involved in cell metabolism^33^. In addition, microgravity leads to hippocampal-dependent learning and memory impairment in animals^34^.

Cytokines are important mediators of both innate and adaptive immunity. They are produced by resident immune cells in the CNS, peripheral tissues, and in circulation^35,36^. There is emerging evidence that cytokines contribute to the detrimental effects of isolation triggered by disease or injury^37,38^. For example, hippocampal expression of interleukin-1 beta (IL-1β) is significantly increased 24 h after ischemia in isolated mice compared to untreated controls, but not in pair-housed mice^37,38^. In humans as well as animals, both spaceflight and simulated microgravity (bedrest) can lead to changes in cytokine expression^10,12,39–42^.

Therefore, in this study, we tested the hypothesis that isolation and simulated microgravity lead to altered hippocampal cytokine expression via mitochondrial ROS-related mechanisms. Hippocampal alterations, e.g., oxidative damage, spatial changes, and genetic alterations have been reported in both actual and modeled microgravity^32,43–46^ and linked to behavioral deficits^47^. These higher CNS functions are critical for astronaut performance during extended periods of spaceflight. Therefore, the hippocampus was selected as the focus of the current study. We found that quenching mitochondrial ROS mitigated the effects of both simulated microgravity and isolation in the hippocampus. Thus, these findings highlight the importance of mitochondrial ROS mechanisms in CNS responses to these stressors, thereby providing a rationale for testing possible use of antioxidants to mitigate CNS risks associated with long-term space exploration as well as bedrest and isolation on Earth.

## Results

### Effects of isolation, microgravity and their combination on hippocampal cytokine levels

**Fig. 1:**
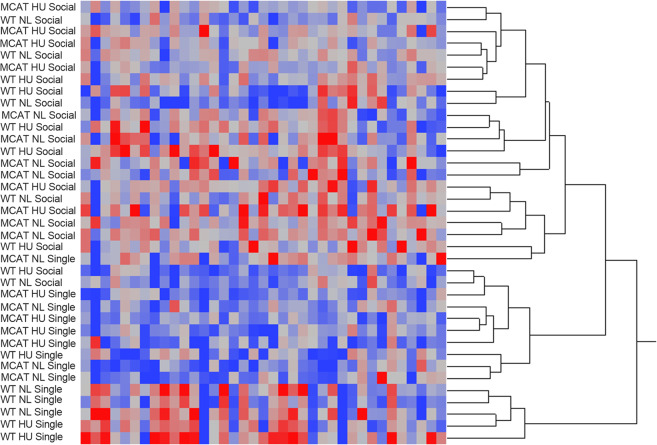
Heatmap showing expression of a panel of 44 cytokines in hippocampus and hierarchical clustering analysis of eight experimental groups. Except for a few samples, clustering generally occurred by housing environment (single or social). Deeper shades of red: higher cytokine levels, deeper shades of blue: lower cytokine levels, off-white: no change in expression.

We performed unbiased hierarchical clustering of cytokine protein expression to gain insight into the impacts of our main experimental variables, loading state (NL: normally loaded or HU), housing environment (single or social housing), and genotype (WT: wild type or MCAT) on the overall cytokine milieu in the hippocampus (Fig. 1). At the highest hierarchical level, samples generally clustered by housing conditions (single or social). Samples within the single housed cluster, formed sub-clusters mostly based on genotype (WT or MCAT), which was not observed in the social housed cluster. Loading state (NL or HU) did not appear to contribute to observed clustering of cytokine expression.

**Fig. 2:**
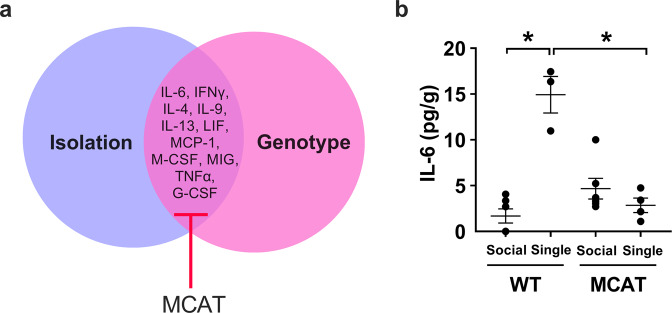
Cytokines showing higher expression in hippocampus due to isolation and mitigation in MCAT mice. **a** Eleven out of 44 cytokines were upregulated in wild-type single housed vs social-housed mice and mitigated in MCAT single housed mice. **b** Representative graph (IL-6) of one of the 11 cytokines elevated due to isolation and mitigated in MCAT mice. Here and in succeeding dot plots, the longer horizontal line in the dot plot corresponds to the group mean while the shorter horizontal lines depict the standard error (SE). Sample sizes are WT NL social (*n* = 6), WT NL single (*n* = 3), MCAT NL social (*n* = 6), and MCAT NL single (*n* = 4). Some data points overlap. A fit model was generated to include housing state, genotype, loading state, and their pairwise interactions. Interaction effects between housing state and genotype were assessed at *p* < 0.05. *Statistically significant at *p* < 0.05 by Tukey post hoc test.

We first examined the effects of housing environment in all groups (Fig. 2). A comparison between single and social housed mice revealed marked effects of isolation in the hippocampus. Eleven out of 44 cytokines were elevated in wild-type, single housed mice versus the social-housed group (Fig. 2a and Supplementary Table 3). These effects were mitigated in MCAT mice. IL-6 (Fig. 2b and Supplementary Table 3) is provided as a representative example of the expression pattern evident amongst these 11 cytokines.

**Fig. 3:**
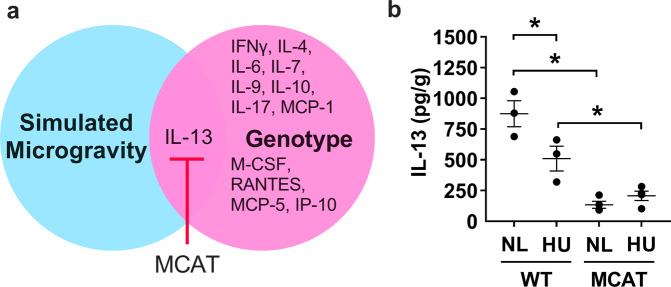
Effect of combined isolation and simulated microgravity and the impact of MCAT genotype on hippocampal cytokine expression (single housed groups only). **a** Diagram showing the effects of simulated microgravity and genotype on hippocampal cytokine expression in single housed animals. **b** IL-13 protein levels showing downregulation by HU and further decreased by MCAT genotype. WT NL (*n* = 3), WT HU (*n* = 3), MCAT NL (*n* = 4), and MCAT HU (*n* = 4). Some data points overlap. *Statistically significant at *p* < 0.05 by two-way ANOVA and Tukey post hoc test.

Next, we focused on the effects of simulated microgravity and genotype (as main factors and their interactions) on hippocampal cytokine expression in single housed mice (Fig. 3 and Supplementary Table 4). When exploring the effect of genotype (main factor) in single housed mice, we found differences in cytokine expression (Fig. 3a), with 13 out of 44 cytokines showing reduced protein levels in single housed MCAT versus wild-type mice (Supplementary Table 5). HU downregulated IL-13 in WT mice. Interestingly, IL-13 levels were lower in MCAT NL mice than in WT NL mice. HU did not appear to cause any further decreases in IL-13 levels of MCAT animals, suggesting mitigation of cytokine changes (Fig. 3b and Supplementary Table 4).

**Fig. 4:**
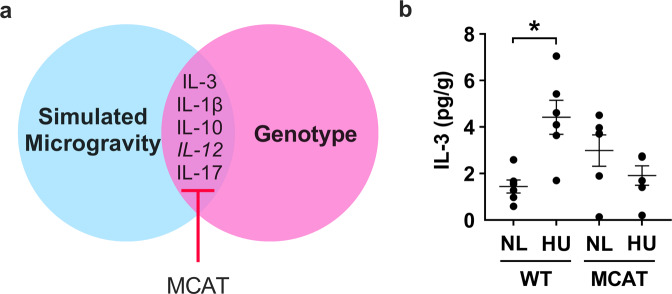
Effects of simulated microgravity (without isolation) and genotype on the expression of hippocampal cytokines (social housed groups only). **a** Five hippocampal cytokines were differentially expressed in social housed HU versus NL mice and changes mitigated in MCAT mice. Cytokine in italics (IL-12) was downregulated while the rest were upregulated. **b** Representative graph showing upregulated hippocampal cytokine in social housed HU vs NL mice and mitigation in MCAT mice. Sample size is *n* = 6/group. Some data points overlap. *Statistically significant at *p* < 0.05 by two-way ANOVA and Tukey post hoc test.

In social housed animals, simulated microgravity and transgene expression caused changes in the expression of five hippocampal cytokines (Fig. 4a). Four out of these five cytokines (IL-3, IL-1β, IL-10, and IL-17) were upregulated in HU versus NL mice, and mitigated in MCAT mice (Supplementary Table 6). Results for IL-3 are shown as a representative example of the upregulated cytokines (Fig. 4b). In contrast, (IL-12) was downregulated and this effect also was mitigated in MCAT animals (Supplementary Table 6). Some of the cytokines showed relatively low expression levels (e.g., IL-6, IL-1β, refer to Supplementary Table 1 for full list of cytokines) relative to other cytokines in the panel. These results are consistent with values reported in other studies that suggest biologically relevant effects at these levels of expression^48,49^. Table 1 summarizes the results of cytokine analysis of hippocampus.

**Table 1:** Summary of the response of each genotype to isolation, simulated microgravity, or both.

Genotype	Treatment	Hippocampus	Plasma
Wild type	Isolation	Upregulation of 11 cytokines (IL-6, IFN-γ, IL-4, IL-9, IL-13, LIF, MCP-1, M-CSF, TNF-α, MIG, G-CSF); Fig. 2	Upregulation of two cytokines (Eotaxin, MDC); Fig. 7
			Downregulation of corticosterone levels^a^
MCAT	Isolation	Mitigation of cytokine changes; Fig. 2	No mitigation of cytokine changes; Fig. 7
			No change in corticosterone levels
Wild type	Simulated microgravity	Upregulation of four (IL-3, IL-1β, IL-10, IL-17) and downregulation of one cytokine(s) (IL-12); Fig. 4	Upregulation of one cytokine (IL−20); Fig. 6
			Upregulation of corticosterone levels
MCAT	Simulated microgravity	Mitigation of cytokine changes; Fig. 4	Mitigation of cytokine changes; Fig. 6
			Mitigation of corticosterone changes
Wild type	Isolation + Simulated microgravity	Downregulation of one cytokine (IL-13); Fig. 3	Upregulation of two (IL−20, IFN-β1) and downregulation of one cytokine(s) (MDC); Fig. 5
			Upregulation of corticosterone levels
MCAT	Isolation + Simulated microgravity	Mitigation of cytokine change; Fig. 3	Mitigation of cytokine changes; Fig. 5
			Mitigation of corticosterone changes

The main figure numbers corresponding to the summarized cytokine results are also included in the table. “Mitigation of cytokine change(s)” (by MCAT) pertains to mitigation of all the cytokine changes observed in the corresponding wild-type group.

^a^Only in NL groups; Corticosterone levels in HU groups are not further reduced by isolation.

### Effects of isolation, microgravity, and their combination on plasma cytokine levels

**Fig. 5:**
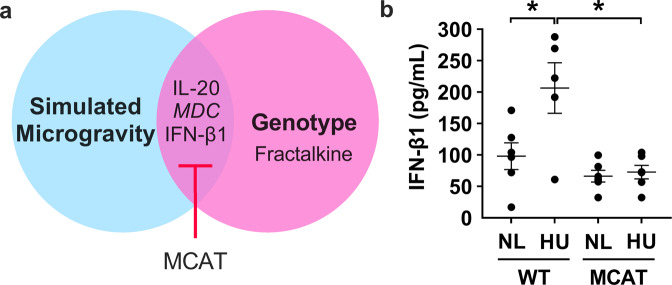
Effects of combined isolation and simulated microgravity and the impact of MCAT genotype on plasma cytokine expression (single housed groups only). **a** Diagram showing the effect of simulated microgravity and genotype on plasma cytokine expression in single housed animals. Three cytokines were differentially expressed in plasma of single housed HU versus NL mice and mitigated in MCAT HU mice. MDC was downregulated (italics) while IL-20 and IFN-β1 were upregulated. **b** Representative graph showing IFN-β1, one of the two cytokines elevated by HU and mitigated in MCAT mice. NL WT (*n* = 6), HU WT (*n* = 5), NL MCAT (*n* = 6), and U MCAT (*n* = 6). Some data points overlap. *Statistically significant at *p* < 0.05 by two-way ANOVA and Tukey post hoc test.

The same 44-cytokine protein expression analysis was performed on blood plasma to assess possible system-wide effects of simulated microgravity and isolation. Cytokine expression patterns in the plasma differed markedly from those observed in the hippocampus. Figure 5a shows the effects of simulated microgravity and genotype on cytokine plasma expression in single housed mice. In single housed mice, HU upregulated expression of IL-20 and IFN-β1, whereas these effects were not observed in MCAT mice (Supplementary Table 7). In contrast, HU downregulated MDC (macrophage-derived chemokine), which also was mitigated in MCAT mice (Fig. 5b and Supplementary Table 7). Fractalkine showed a genotype effect (Supplementary Table 8).

**Fig. 6:**
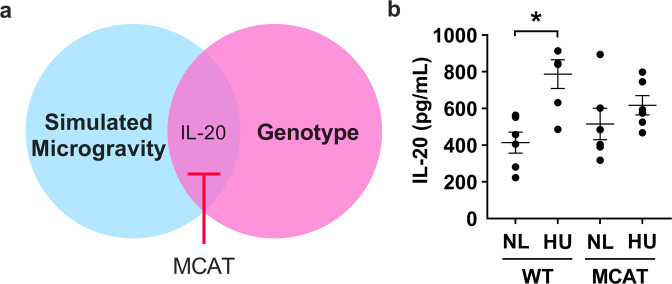
Effect of simulated microgravity (without isolation) and genotype on the expression of plasma cytokines (social housed groups only). **a** One cytokine, IL-20, was elevated due to HU in plasma of social housed mice and this effect is mitigated in MCAT animals. **b** Graph showing protein expression of IL-20 in plasma of WT and MCAT animals exposed to HU. Sample size is *n* = 6/group. Some data points overlap. *Statistically significant at *p* < 0.05 by two-way ANOVA and Tukey post hoc test.

In social housed groups (excluding isolation as a factor), there was a twofold increase in IL-20 protein levels in the plasma of HU mice compared to NL controls. This effect was mitigated in MCAT mice (Fig. 6 and Supplementary Table 9). Within the 44 cytokines tested, isolation showed less impact on plasma cytokine expression levels than observed in the hippocampus.

**Fig. 7:**
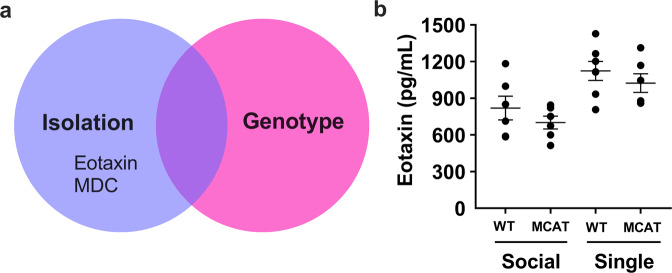
Effect of isolation on plasma cytokines. **a** Isolation elevates the levels of Eotaxin and MDC in plasma and are not mitigated in MCAT mice. **b** Representative graph (Eotaxin) showing the effects of isolation on plasma cytokines and no mitigation in MCAT mice. WT NL social (*n* = 6), WT NL single (*n* = 6), MCAT NL social (*n* = 7), and MCAT NL single (*n* = 6). Some data points overlap. A fit model was created and included housing state, genotype and loading state, and their pairwise interactions. Interaction effects between housing state and genotype were assessed at *p* < 0.05.

Isolation affected only two cytokines in plasma—Eotaxin and MDC (Fig. 7). These cytokines were upregulated in single housed animals and unchanged in MCAT mice (Supplementary Table 10). Refer to Table 1 for a summary of results from plasma cytokine analysis.

### Assessment of corticosterone levels in plasma

To begin investigating possible underlying stress-related mechanisms, we measured plasma corticosterone levels in all groups (Supplementary Fig. 2 and Supplementary Table 11). Corticosterone levels were generally higher in the social groups compared to their corresponding single housed groups (only WT single vs WT social housed HU groups showed no differences). We previously reported that HU of wild-type animals led to increased corticosterone levels^13^. In the current study, we found that single housed MCAT mice were protected from HU-induced increases in corticosterone levels. Interestingly, social housed MCAT NL mice had higher corticosterone levels versus social housed WT NL mice. However, HU in social housed MCAT mice did not lead to additional increases in corticosterone levels relative to the social MCAT NL group. Refer to Table 1 for a summary of corticosterone results.

### Measurements of oxidative damage in hippocampus

Changes in inflammatory status have been linked to differences in oxidative damage^50,51^. To determine whether isolation or simulated microgravity caused oxidative damage in the hippocampus, we measured 4-hydroxynonenal (4-HNE) adduct levels. Single housed WT animals generally showed higher levels of 4-HNE compared to social housed WT mice (Supplementary Fig. 3A, and Supplementary Table 12). In contrast, 4-HNE levels of single versus social housed MCAT groups were generally comparable. Further, no HU effect was observed (Supplementary Table 13).

## Discussion

Although not in complete solitude, human crew in deep space missions will experience prolonged exposure to environments isolated from Earth and larger social groups. Using a rodent model, we assessed the consequence of isolation alone or in combination with simulated microgravity on the CNS cytokine milieu, a key component of the neuroimmune response. In addition, we sought to determine whether mitochondrial ROS signaling contributed to cytokine responses in the CNS focusing on the hippocampus. Spaceflight is known to cause dysfunction in a variety of tissues^52–57^. Various regions of the CNS are negatively impacted by microgravity and its simulations. For example, 14 days of HU increased acetylcholine activity in both hippocampus and cortex, which was linked to inferior behavior performances^47,58^. Our results suggest that isolation is an important stimulus that can evoke changes in the cytokine milieu of the hippocampus, with 11 of 44 immune-modulating cytokines tested showing increased protein expression due to isolation. We also found that compared to social housed mice, isolation increased 4-HNE, an indicator of oxidative damage. Together, these findings indicate that isolation can alter aspects of CNS immune signaling and lead to increased oxidative damage in the CNS.

Changes in cytokine signaling in the CNS have been linked to neuroinflammation, neurocognitive decline, reduced ability to recover from brain injuries as well as neuroprotective effects. For example, genetic deletion of IL-6 leads to reduced exploratory behavior and decreased anxiety^59^. Deletion of IL-2 in mice leads to cognitive deficits that resemble aspects of Alzheimer’s disease^60^ while disruption of Toll-like receptor signaling by knockout of TLR4 protects against age-related cognitive decline due to neuroinflammation and apoptosis^61^. Taken together, findings from our study and others suggest that isolation poses a risk to CNS health.

Isolation and HU led to discrete sets of differentially expressed cytokines in hippocampus, suggesting different immunoregulatory mechanisms invoked by each of these stressors. Collectively, the cytokines differentially expressed in response to HU or isolation as single factors are known to play roles in immune regulation, inflammatory disease and also are impacted by spaceflight. (Refer to ref. ^62^ for a recent review on the functions of interleukins and other cytokines). For example, circulating IL-20 protein levels (elevated by HU in our current study) are also increased at the early stages of rheumatoid arthritis and decreased after 6 months of treatment^63,64^. Further, IL-3 protein levels are greater in splenocytes and thymocytes from space-flown rats than ground controls^65^. In peripheral blood mononuclear cells obtained from astronauts onboard the ISS, IL-10 is reduced following lipopolysaccharide stimulation compared to controls^7^. Thus, our observations that simulated weightlessness and isolation provoke expression of discrete cytokine repertoires is consistent with our previous findings on immune cell responses using these models^13^.

At the timepoint examined in this study, isolation had a more pronounced impact than HU on the expression of cytokines in the hippocampus, as indicated by differential expression of 11 cytokines as a result of isolation (single housed compared to social housed NL groups) versus 5 cytokines due to HU (social housed HU compared to NL groups). In addition, HU under single and social housed conditions resulted in distinct sets of differentially expressed cytokines in the hippocampus. Taken together, our findings support the hypothesis that simulated weightlessness and isolation activate discrete molecular and physiological mechanisms to regulate cytokine production.

We found that cytokine responses to spaceflight stressors in the hippocampus versus circulation were also discrete under the experimental conditions used in this study. Further, isolation-induced cytokine responses were more extensive in the hippocampus compared to the plasma, based on the higher number of cytokines affected (11 and 3, respectively). Although only a single timepoint (30 days) was assessed in this study, our findings raise the possibility that changes in cytokine levels in the circulation may not mirror those in the brain during spaceflight. The 30 day HU period is considered a chronic exposure paradigm given that it accounts for 4% of an animal’s lifespan (assuming a 2 year lifespan). For perspective, a trip to Mars can take about two years, corresponding to 2.5% of a human lifespan (assuming 80 years as the average human lifespan).

Our study is the first to implicate mitochondrial ROS in the CNS and in circulating cytokine levels brought about by combined isolation and simulated microgravity. MCAT mice exhibited protection from the hippocampal cytokine changes caused by isolation, simulated microgravity, and in combination. In plasma, HU-induced changes in cytokine expression were mitigated in MCAT mice. In the hippocampus of single housed MCAT mice, both IL-6 and TNFα show reduced expression relative to housing matched wild-type controls. Both of these cytokines are known to play a role in inflammation (chronic and acute) and in autoimmunity. In the same mice cohort we also found that HU elevated circulating neutrophils and neutrophil to lymphocyte ratio (NLR), suggesting increased inflammation while this effect was mitigated in MCAT mice^66^. Taken together, our findings are consistent with our hypothesis, highlighting the important role of ROS in modulating systemic and neuroimmune responses to simulated spaceflight.

Our study contributes new information on the regulation of cytokine expression in the hippocampus in MCAT mice, a widely used genetic model for quenching mitochondrial ROS and studying diseases related to aging. MCAT mice have increased mean and maximum lifespan^15^, enhanced hippocampal spatial learning and memory, reduced contextual fear conditioning, delayed lung cancer onset^67^, and delayed age-related cardiac and ocular pathologies^15^. Some of the downregulated cytokines we observed in MCAT mice contribute to the cancer microenvironment (e.g., IL-6, INFγ, M-CSF, TNFα)^67^ and other metabolic diseases. For example, MCP-1 and IL-6 contribute to the development of abnormal glucose and lipid metabolism^68–70^. Future studies could be conducted to test ROS-mediated modulation of the cytokine milieu as a potential mechanism for the resistance of MCAT mice to the abovementioned aging-related pathologies. In addition, our findings and those of others provide a rationale for future investigations on ROS quenching as a potential approach to mitigate inflammaging, the chronic low-grade inflammation associated with aging^21^.

As we have reported previously, HU increased circulating corticosterone levels of WT mice after 30 days regardless of housing environment^13^. This effect was mitigated in MCAT single housed mice. In social housed NL animals, corticosterone levels were generally higher compared to the corresponding single housed group. The underlying basis for the higher corticosterone levels in these social housed mice versus single housed mice is unclear. Social hierarchy may exist between pair-housed female mice^71^, which may elevate the average corticosterone levels in paired housing versus single housing conditions. The social MCAT NL and HU groups had comparable corticosterone levels. HU in social housed MCAT mice did not lead to further increases in corticosterone levels possibly due to maximum upregulation of the HPA axis and/or a protective effect by the MCAT genotype. Interestingly, increased plasma corticosterone levels were observed in social housed mice even as plasma levels of an oxidative damage marker (4-HNE) and a subset of inflammatory cytokine protein levels in the hippocampus generally decreased. This suggests that elevated stress-associated hormones need not always coincide with pro-inflammatory and pro-oxidative states. This also raises the possibility that the increase in corticosterone levels in social housed groups is not a pathological response. The underlying mechanisms for the inverse relationship of corticosterone with both oxidative damage and inflammatory cytokines require further study.

Female mice were selected for this study. Sex-specific differences in rodent responses to isolation can occur^72,73^. In humans, males and females show some differences in their responses to confinement^74^ and bedrest, another microgravity analog^75,76^. Therefore, future investigations are needed to assess the sex-dependent effects of isolation and microgravity as well as any possible differences in oxidative stress responses. In summary, isolation and long-term simulated microgravity via HU altered cytokine expression levels in both the hippocampus and plasma. These effects in the brain were mitigated in MCAT mice, implicating an important role for mitochondrial ROS in aspects of the neuroimmune response to microgravity and isolation. Taken together, our findings and those of others provide a rationale for the use of antioxidant-based approaches to address anticipated CNS changes during spaceflight and in situations of isolation and reduced mobility on Earth.

## Materials and methods

### Animals and strains

Female wild-type C57BL/6NJ and MCAT transgenic mice were included in this study. MCAT mice overexpress human catalase localized to the mitochondria^15^. Wild type (WT) and MCAT mice for use in the experiments were generated at NASA Ames Research Center (ARC) by crossing male MCAT mice, B6.Cg-Tg (CAG-OTC/CAT) 4033Prab/J with female C57BL/6NJ mice (from Jackson Laboratory; stocks 016197 and 005304, respectively). Genotyping was performed using the Red’N Amp PCR kit following the manufacturer’s protocol (Millipore-Sigma). All animal procedures were performed in compliance with protocols approved by the Institutional Animal Care and Use Committee at ARC. The approved animal protocols associated with this study are NAS-16-006 and NAS-16-007.

### Hindlimb unloading

Sixteen-week-old female wild-type C57BL/6NJ and MCAT mice on C57BL/6NJ background were assigned to one of eight experimental groups, and housed either individually (single housed) or in pairs (social housed) (Supplementary Fig. 1). Females were selected for this study in part because the majority of rodent studies to date on simulated and actual weightlessness have used females. The wild-type groups in this study were the same animals used in our previous report which focused on the assessment of musculoskeletal outcomes and flow cytometry-based immune cell profiling in response to isolation^13^. The traditional NASA Ames HU cage system was used for single housed HU animals^77^, while normally loaded (NL) single housed controls were maintained single housed in standard vivarium cages. A separate validation experiment confirmed that NL mice housed in standard vivarium cages or in HU cages did not differ in body weights, plasma corticosterone levels, soleus mass, bone structure, and immune cell populations in peripheral blood (data not shown). A custom caging system as we had previously described^13^, was used for social housed, HU mice (pairs). Social housed, NL controls were maintained in standard vivarium cages in pairs as controls for the social HU mice. Animals assigned to HU groups were acclimated for 3 days in HU cages but without unloading, prior to the onset of HU. Room temperature was maintained at a range of 23–24 °C with a 12-h light: 12-h dark cycle. Food and water were provided ad libitum. Nestlests (Ancare) were provided as enrichment for the animals. Animal weights and food consumption were monitored throughout the duration of the experiment. Animals were euthanized 30 days after the onset of HU via CO_2_ inhalation followed by cervical dislocation.

### Sample collection and analysis

HU was initiated across three consecutive days with equal numbers of wild-type and MCAT mice (similar ages) on any given day to accommodate the large number of animals in this study. All experimental groups experienced the same length of treatments (e.g., HU). Consequently, dissections were performed across 3 consecutive days.

The left brain hemisphere was flash frozen and a tissue biopsy (1.20 mm Harris micro punch) from the hippocampus was performed inside a cryotome chamber set at −20 °C to avoid thawing. Hippocampal tissue was then homogenized in mild lysis buffer (Tris 50 mM, NaCl 150 mM, Igepal 1%), Protease Inhibitors (Millipore-Sigma)^78^. Samples were then centrifuged at 4 °C at 1000 × *g* for 10 min and supernatants aliquoted and frozen at −80 °C until analysis.

Cytokine protein abundance in hippocampal homogenates were analyzed using a Mouse Cytokine/Chemokine Array 44-Plex-MD44 (EVE Technologies), and concentration standards run for each cytokine. IL-3 cytokine expression was validated by ELISA (Abcam, Cat# ab113345-IL-3). Hippocampal cytokine levels were normalized to total protein content as determined by BCA assay.

Blood was collected from the vena cava immediately after euthanasia. Undiluted plasma was analyzed for cytokine protein abundance as employed for hippocampal homogenates.

### Statistical analysis for cytokine analysis

Statistical analysis of cytokine expression was performed using JMP (SAS), Version 14.0.0. Equal variance was first evaluated by Levene’s test and normality assessed by Shapiro–Wilk goodness of fit test. If equal variance and normality were met, a fit model was performed taking into account the three main effects: loading state (NL or HU), housing (single or social), and genotype (WT or MCAT) and comparisons between the main effects done two at a time (two-way ANOVA) (Supplementary Fig. 1). A two-way ANOVA was selected since we were interested in understanding how one factor interacted with one other factor. The threshold of statistical significance for both main factor and interaction effects was set at *p* < 0.05. If interaction effects were evident, a Tukey post hoc test was performed at *p* < 0.05. If a main effect was evident, a Dunnett’s test was applied to determine differences between groups of that main effect. A two-way ANOVA also was conducted to evaluate the effects of loading state (NL or HU), genotype (WT or MCAT), and loading state × genotype on the four single housed groups (Supplementary Fig. 1). Similarly, a two-way ANOVA was performed on the four social housed groups (Supplementary Fig. 1). For datasets with unequal variance and/or non-normal distributions, a non-parametric Wilcoxon all pairs test was performed with statistical significance set at *p* < 0.05. Refer to Supplementary Tables 1–13 for more info on *p* values and summary of the statistical testing applied in this study.

### Depiction of cytokine results: Venn diagrams

On the basis of results derived from two-way ANOVA, statistically significant differences in cytokine abundance were summarized in Venn diagrams. As described specifically in each figure and legend, each circle in the diagram represents groups combined according to the various main factors, i.e., loading state (NL or HU, described as Simulated Microgravity effects), or housing (single or social, described as Isolation effects), or genotype (WT or MCAT, described as Genotype effects), with specific cytokines affected by the factors listed within each circle. The intersect of two circles depicts which cytokines were affected by both main factors. A crossbar abutting the intersect of the circles depicts findings that differences in cytokine levels common to both main factors were mitigated in MCAT mice relative to WT mice.

### Assays for oxidative damage

Hippocampal homogenates were analyzed for ROS and related products. 4-Hydroxynonenal (4-HNE) adduct was measured by OxiSelect HNE Adduct Competitive ELISA (Cell Biolabs, Cat# STA-838).

### Assay for plasma corticosterone

Plasma corticosterone was analyzed in 1:100 diluted plasma by ELISA (Abcam, Cat# ab108821).

### Statistical analysis for corticosterone and assays for oxidative damage

Statistical analysis as performed using JMP (SAS), Version 14.0.0. Equal variance was first evaluated by Levene’s test and normality assessed by Shapiro–Wilk goodness of fit test. For 4-HNE results, a linear fit model was created using a least squares approach, and involved the three main variables (loading, housing environment, genotype) and their pairwise interactions (2 × 2 × 2 analysis of variance, ANOVA). If interaction effects were observed (*p* < 0.05), a Tukey post hoc test was performed. For corticosterone results, a non-parametric Wilcoxon all pairs test was performed with statistical significance set at *p* < 0.05.

### Reporting summary

Further information on research design is available in the Nature Research Reporting Summary linked to this article.

## Supplementary information

Supplementary information

Reporting Summary

## Data Availability

All data generated or analyzed during this study are included in this published article and its supplementary information files.
