# Supplementary material for: Overexpression of catalase in mitochondria mitigates changes in hippocampal cytokine expression following simulated microgravity and isolation
Source: NPJ Microgravity. 2021 Jul 6;7:24. doi: 10.1038/s41526-021-00152-w (PMC8260663; doi:10.1038/s41526-021-00152-w)
Supplement: Supplementary file 1 — Supplementary information [file 41526_2021_152_MOESM1_ESM.pdf]

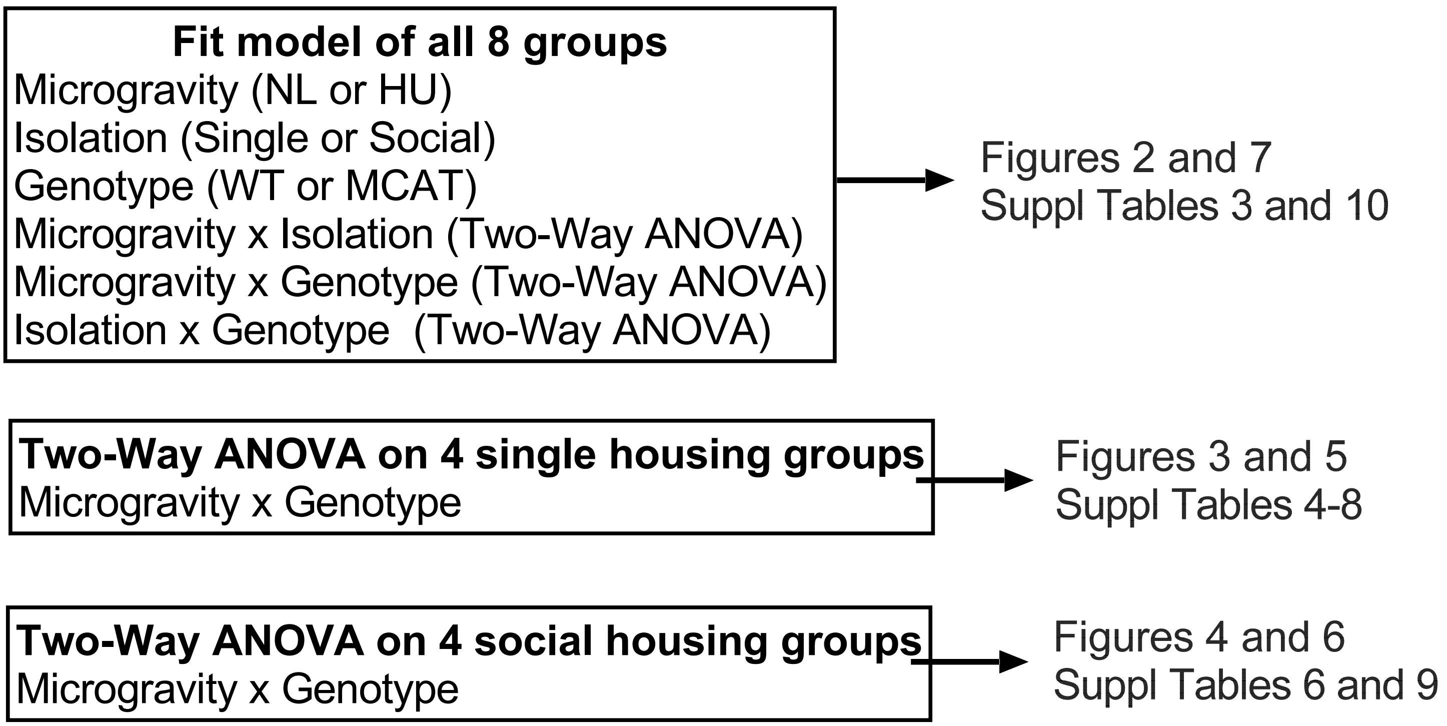

Supplementary Figure 1. Schematic diagram of statistical tests applied in this study. A robust main effect of housing was observed using the fit model. Hence, we performed two separate two-way ANOVA on the single and social housed groups to observe any subtle differences from the other two main effects and their interactions which could have been subsumed by the housing (isolation) effect.

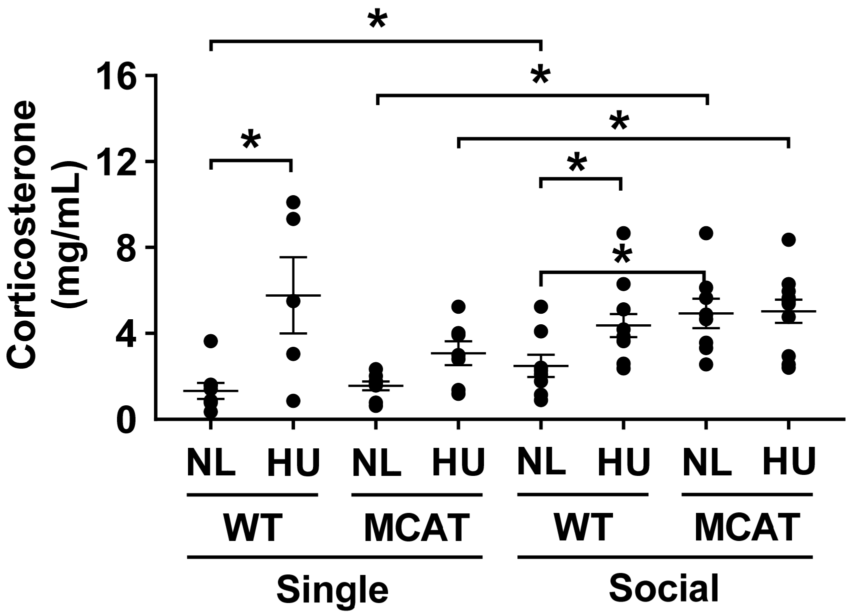

Supplementary Figure 2. Plasma corticosterone levels across experimental groups. Corticosterone levels of the wild type groups have been reported previously [13]. WT NL single (n=8), WT HU single (n=5), MCAT NL single (n=8), MCAT HU single (n=7), WT NL social (n=8), WT HU social (n=11), MCAT NL social (n=8), and MCAT HU social (n=11). Some data points overlap. *Statistically significant at $p<0.05$ by non-parametric Wilcoxon test between all groups.

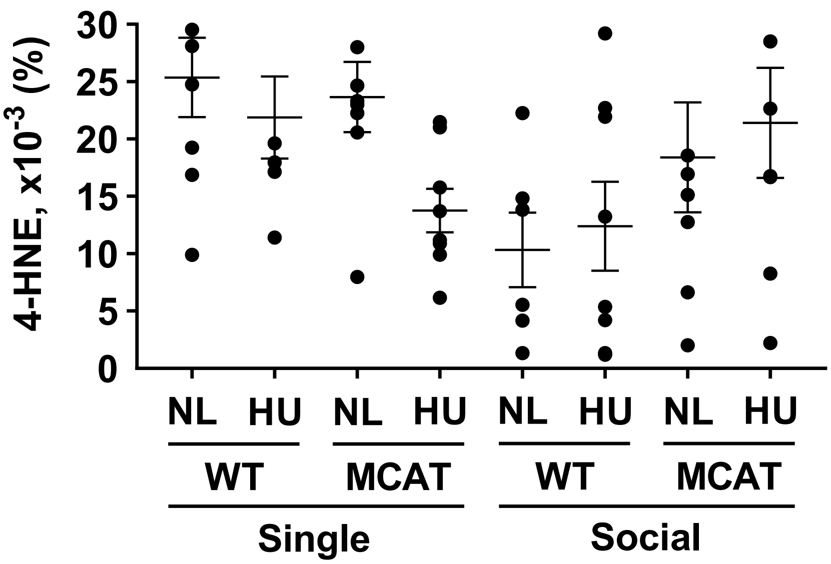

Loading	0.439
Housing	**0.043**
Genotype	0.464
Loading x Genotype	0.610
Housing x Genotype	**0.014**
Housing x Loading	0.073

Supplementary Figure 3. Percentage of 4-HNE protein levels in the hippocampus of WT and MCAT animals that were single or social housed, normalized to total protein content. WT NL single (n=8), WT HU single (n=6), MCAT NL single (n=8), MCAT HU single (n=8), WT NL social (n=6), WT HU social (n=8), MCAT NL social (n=8), and MCAT HU social (n=8). Some data points overlap. Threshold of statistical significance at $p<0.05$ by two-way ANOVA.

Supplementary Table 1. Expression levels of hippocampal cytokines per group expressed in pg/g tissue. Values shown are means, standard deviation (SD) and standard error (SE). Sample sizes are: WT NL single (n=3),WT HU single (n=3), MCAT NL single (n=4), MCAT HU single (n=4), WT NL social (n=6), WT HU social (n=6), MCAT NL social (n=6) and MCAT HU social (n=6). These groups are collapsed into two main factors (housing and genotype) in the statistical analysis shown in Figure 2. Superscript "L" indicates cytokines that had lower levels of expression (close to lowest datapoint in standard curve) but were included due to statistically significant differences across groups. Samples that showed undetectable cytokine levels were assigned a value of zero. These values were included in the calculation of means, standard errors and standard deviations.

**Group Mean (pg/g)**	**Eotaxin**	**G-CSF**	**IFNy**	**IL-1a**	**IL-1B**	**IL-2**
WT NL Single (n=3)	51.582	9.748	162.263	1351.628	160.193	2188.558
WT HU Single (n=3)	77.695	6.891	162.846	1298.174	164.292	1788.241
MCAT NL Single (n=4)	50.726	0.000	67.569	1108.710	187.334	1820.985
MCAT HU Single (n=4)	42.038	2.578	56.018	1252.311	212.166	1907.173
WT NL Social (n=6)	54.455	1.366	70.500	1707.017	176.485	1660.514
WT HU Social (n=6)	50.856	2.831	78.214	2009.429	276.195	1869.611
MCAT NL Social (n=6)	59.085	4.102	90.374	1815.366	222.934	2102.288
MCAT HU Social (n=6)	66.221	2.400	89.132	1798.477	204.512	2149.208

**Group SE**	**Eotaxin**	**G-CSF**	**IFNy**	**IL-1a**	**IL-1B**	**IL-2**
WT NL Single	5.423	1.313	25.672	100.540	51.165	119.130
WT HU Single	16.226	0.904	28.362	250.540	75.297	278.241
MCAT NL Single	2.845	0.000	9.923	165.408	49.253	180.833
MCAT HU Single	6.182	2.578	7.798	129.666	14.571	98.506
WT NL Social	7.037	0.714	12.336	61.613	22.349	135.332
WT HU Social	8.917	1.335	11.257	186.242	34.353	77.483
MCAT NL Social	5.402	2.069	8.312	207.103	31.091	152.154
MCAT HU Social	6.044	1.545	6.639	61.906	23.943	245.451

**Group SD**	**Eotaxin**	**G-CSF**	**IFNy**	**IL-1a**	**IL-1B**	**IL-2**
WT NL Single	9.393	2.274	44.466	174.141	88.620	206.339
WT HU Single	28.105	1.566	49.124	433.947	130.418	481.928
MCAT NL Single	5.691	0.000	19.847	330.816	98.506	361.665
MCAT HU Single	12.365	5.155	15.597	259.332	29.142	197.013
WT NL Social	17.236	1.748	30.218	150.921	54.744	331.494
WT HU Social	21.841	3.269	27.574	456.198	84.148	189.795
MCAT NL Social	13.232	5.068	20.361	507.297	76.157	372.700
MCAT HU Social	14.806	3.785	16.263	151.637	58.648	601.229

Supplementary Table 1
continued

**Group Mean (pg/g)**	**IL-3^{L}**	**IL-4^{L}**	**IL-6**	**IL-7**	**IL-9**	**IL-10**
WT NL Single (n=3)	1.018	3.571	14.922	35.651	36814.627	173.874
WT HU Single (n=3)	0.410	4.106	12.011	34.468	35701.514	186.732
MCAT NL Single (n=4)	0.782	0.000	2.854	27.083	21853.399	101.573
MCAT HU Single (n=4)	0.711	0.968	2.139	13.484	22496.981	73.968
WT NL Social (n=6)	1.443	1.704	1.686	8.631	18460.541	90.684
WT HU Social (n=6)	4.418	1.075	4.591	29.795	20196.341	131.791
MCAT NL Social (n=6)	2.987	2.239	4.668	21.398	22731.193	161.818
MCAT HU Social (n=6)	1.913	2.083	3.010	20.374	25455.140	122.614

**Group SE**	**IL-3**	**IL-4**	**IL-6**	**IL-7**	**IL-9**	**IL-10**
WT NL Single	0.519	0.431	1.997	1.954	5389.594	29.209
WT HU Single	0.210	1.326	2.406	6.291	4817.570	26.734
MCAT NL Single	0.492	0.000	0.790	7.687	3936.835	23.577
MCAT HU Single	0.389	0.382	0.909	3.004	1664.734	17.160
WT NL Social	0.280	0.647	0.774	5.128	2386.454	8.647
WT HU Social	0.731	0.309	1.099	8.256	2423.211	17.820
MCAT NL Social	0.674	0.752	1.125	6.736	2526.618	17.051
MCAT HU Social	0.417	0.327	1.259	5.455	2811.431	10.041

**Group SD**	**IL-3**	**IL-4**	**IL-6**	**IL-7**	**IL-9**	**IL-10**
WT NL Single	0.899	0.746	3.459	3.384	9335.051	50.591
WT HU Single	0.363	2.296	4.167	10.896	8344.276	46.304
MCAT NL Single	0.984	0.000	1.580	15.373	7873.671	47.154
MCAT HU Single	0.778	0.764	1.818	6.009	3329.469	34.320
WT NL Social	0.686	1.585	1.896	12.560	5845.595	21.181
WT HU Social	1.792	0.756	2.692	20.224	5935.630	43.650
MCAT NL Social	1.651	1.843	2.754	16.501	6188.924	41.765
MCAT HU Social	1.022	0.800	3.083	13.361	6886.572	24.595

Supplementary Table 1 continued

Group Mean (pg/g)	IL-12	IL-12	IL-13	IL-15	IL-17	IP-10
WT NL Single (n=3)	0.000	34.434	874.459	22.140	15.286	163.536
WT HU Single (n=3)	0.000	38.621	509.754	26.771	10.336	264.640
MCAT NL Single (n=4)	0.936	41.310	133.873	36.291	8.786	121.392
MCAT HU Single (n=4)	4.686	33.985	206.917	38.127	8.568	102.347
WT NL Social (n=6)	19.793	33.679	134.699	36.496	9.138	157.448
WT HU Social (n=6)	28.090	58.146	240.443	55.642	12.147	217.487
MCAT NL Social (n=6)	33.945	59.694	198.499	128.142	14.434	139.194
MCAT HU Social (n=6)	42.300	43.090	212.913	49.425	12.311	139.225

Group SE	IL-12	IL-12	IL-13	IL-15	IL-17	IP-10
WT NL Single	0.000	11.034	105.707	1.794	0.199	31.752
WT HU Single	0.000	6.366	100.659	6.620	2.758	49.117
MCAT NL Single	0.844	8.214	28.048	15.296	0.574	17.257
MCAT HU Single	2.879	7.113	37.821	12.079	1.178	4.907
WT NL Social	4.631	4.592	17.686	3.381	1.173	35.603
WT HU Social	7.243	13.099	85.106	9.468	0.801	82.394
MCAT NL Social	5.960	7.221	35.338	88.526	0.974	18.389
MCAT HU Social	9.326	4.427	40.220	9.767	1.347	9.692

Group SD	IL-12	IL-12	IL-13	IL-15	IL-17	IP-10
WT NL Single	0.000	19.111	183.090	3.108	0.344	54.997
WT HU Single	0.000	11.026	174.347	11.467	4.778	85.073
MCAT NL Single	1.689	16.428	56.096	30.592	1.149	34.514
MCAT HU Single	5.758	14.227	75.642	24.157	2.357	9.813
WT NL Social	11.343	11.249	43.322	8.281	2.873	87.209
WT HU Social	17.741	32.087	208.465	23.192	1.962	201.824
MCAT NL Social	14.599	17.688	86.560	216.843	2.385	45.043
MCAT HU Social	22.843	10.843	98.518	23.924	3.299	23.740

Supplementary Table 1
continued

Group Mean (pg/g)	KC	LIF	MCP-1	M-CSF	MIG	MIP-1B
WT NL Single (n=3)	45.868	4.846	3239.241	67.281	347.720	173.281
WT HU Single (n=3)	47.622	6.393	3063.790	73.824	305.420	153.280
MCAT NL Single (n=4)	57.088	5.166	1224.145	51.951	235.008	514.978
MCAT HU Single (n=4)	42.517	1.515	1388.962	41.445	244.083	785.314
WT NL Social (n=6)	58.074	1.148	1033.606	36.485	207.530	839.264
WT HU Social (n=6)	55.016	2.708	1265.585	37.170	241.906	936.148
MCAT NL Social (n=6)	70.229	3.607	1213.761	51.866	262.626	1882.028
MCAT HU Social (n=6)	68.088	3.629	1752.903	50.404	312.438	824.439

Group SE	KC	LIF	MCP-1	M-CSF	MIG	MIP-1B
WT NL Single	6.314	0.316	570.079	14.893	49.838	20.855
WT HU Single	15.254	1.633	809.708	12.612	53.651	52.164
MCAT NL Single	4.207	1.781	324.668	3.945	38.487	148.083
MCAT HU Single	4.825	0.886	232.696	4.134	26.680	93.243
WT NL Social	9.368	0.831	249.854	9.042	28.067	88.017
WT HU Social	4.926	1.044	232.813	5.668	28.053	151.091
MCAT NL Social	4.746	0.900	237.802	6.319	27.863	638.963
MCAT HU Social	6.305	1.169	324.017	5.709	33.288	53.947

Group SD	KC	LIF	MCP-1	M-CSF	MIG	MIP-1B
WT NL Single	10.936	0.547	987.406	25.795	86.321	36.122
WT HU Single	26.421	2.828	1402.456	21.844	92.926	90.351
MCAT NL Single	8.414	3.562	649.337	7.890	76.974	296.167
MCAT HU Single	9.651	1.771	465.393	8.268	53.361	186.486
WT NL Social	22.948	2.035	612.016	22.148	68.750	215.597
WT HU Social	12.065	2.556	570.273	13.884	68.716	370.095
MCAT NL Social	11.624	2.205	582.494	15.479	68.250	1565.132
MCAT HU Social	15.445	2.864	793.675	13.984	81.538	132.143

Supplementary Table 1 continued

Group Mean (pg/g)	MIP-2	RANTES	TNFa[L]	EPO	6Ckine/Exodus 2	Fractalkine
WT NL Single (n=3)	1940.718	40.746	12.700	0.000	2201.004	13126.138
WT HU Single (n=3)	1690.100	29.210	12.135	0.000	7307.682	9997.544
MCAT NL Single (n=4)	1425.015	25.253	3.108	5.653	4793.237	11153.229
MCAT HU Single (n=4)	1702.484	26.250	1.273	5.504	4089.763	6447.159
WT NL Social (n=6)	2659.171	31.559	0.000	6.759	6184.104	7327.448
WT HU Social (n=6)	2662.915	36.634	1.960	8.112	7934.682	9229.097
MCAT NL Social (n=6)	2854.531	40.166	5.116	9.297	5317.047	9334.643
MCAT HU Social (n=6)	2391.728	37.069	0.000	7.527	3929.176	9347.996

Group SE	MIP-2	RANTES	TNFa	EPO	6Ckine/Exodus 2	Fractalkine
WT NL Single	208.424	3.038	1.933	0.000	251.978	3133.952
WT HU Single	344.442	5.157	8.373	0.000	525.183	449.704
MCAT NL Single	216.881	4.201	3.108	0.835	1501.746	322.826
MCAT HU Single	90.992	3.031	1.273	0.307	1764.484	366.177
WT NL Social	157.430	2.108	0.000	0.395	1785.536	932.311
WT HU Social	171.336	2.762	1.244	0.784	1916.181	1017.069
MCAT NL Social	104.126	2.314	1.800	0.943	1327.481	1032.828
MCAT HU Social	160.490	2.768	0.000	0.910	1502.769	1001.867

Group SD	MIP-2	RANTES	TNFa	EPO	6Ckine/Exodus 2	Fractalkine
WT NL Single	361.001	5.261	3.347	0.000	436.439	5428.163
WT HU Single	596.591	8.932	14.503	0.000	909.643	778.911
MCAT NL Single	433.761	8.402	6.216	1.671	3003.492	645.652
MCAT HU Single	181.983	6.062	2.547	0.613	3528.968	732.354
WT NL Social	385.623	5.163	0.000	0.967	4373.653	2283.685
WT HU Social	419.685	6.766	3.047	1.920	4693.665	2491.300
MCAT NL Social	255.056	5.669	4.410	2.310	3251.650	2529.903
MCAT HU Social	393.118	6.780	0.000	2.229	3681.018	2454.062

Supplementary Table 1 continued

Group Mean (pg/g)	IFNB-1	IL-16	MDC	MCP-5	MIP-3a[L]	MIP-3B
WT NL Single (n=3)	50.863	1211.784	520.613	468.845	39.339	7.339
WT HU Single (n=3)	54.209	1275.266	477.061	648.185	35.364	91.268
MCAT NL Single (n=4)	57.438	2870.610	394.665	539.580	30.690	70.729
MCAT HU Single (n=4)	56.502	894.852	337.900	401.038	39.357	19.710
WT NL Social (n=6)	73.677	2165.969	119.078	461.465	54.981	56.977
WT HU Social (n=6)	70.834	1923.203	139.945	647.353	55.221	66.202
MCAT NL Social (n=6)	68.637	2750.356	143.730	451.551	62.531	103.921
MCAT HU Social (n=6)	67.269	1649.891	279.938	435.893	60.111	23.702

Group SE	IFNB-1	IL-16	MDC	MCP-5	MIP-3a	MIP-3B
WT NL Single	1.641	188.956	34.592	79.361	2.726	7.339
WT HU Single	11.317	126.406	121.572	27.592	2.750	22.707
MCAT NL Single	3.582	839.166	47.431	43.599	4.074	49.778
MCAT HU Single	4.435	174.474	50.717	41.008	3.006	11.870
WT NL Social	3.068	449.057	27.157	89.073	2.985	7.836
WT HU Social	3.614	376.102	19.869	294.772	2.680	16.200
MCAT NL Social	8.355	635.606	32.967	68.084	5.859	48.564
MCAT HU Social	6.346	171.426	68.960	35.363	3.608	13.700

Group SD	IFNB-1	IL-16	MDC	MCP-5	MIP-3a	MIP-3B
WT NL Single	2.842	327.281	59.915	137.458	4.722	12.711
WT HU Single	19.602	218.941	210.568	47.791	4.764	39.329
MCAT NL Single	7.164	1678.332	94.862	87.199	8.147	99.556
MCAT HU Single	8.871	348.948	101.434	82.015	6.012	23.741
WT NL Social	7.515	1099.960	66.521	218.184	7.311	19.194
WT HU Social	8.854	921.257	48.669	722.042	6.565	39.681
MCAT NL Social	20.466	1556.911	80.752	166.770	14.352	118.957
MCAT HU Social	15.544	419.907	168.916	86.620	8.838	33.557

Supplementary Table 1 continued

Group Mean (pg/g)	TARC	TIMP-1
WT NL Single (n=3)	119.045	71.105
WT HU Single (n=3)	164.682	160.680
MCAT NL Single (n=4)	128.561	331.468
MCAT HU Single (n=4)	112.632	89.427
WT NL Social (n=6)	106.927	81.997
WT HU Social (n=6)	110.169	79.274
MCAT NL Social (n=6)	115.179	88.427
MCAT HU Social (n=6)	174.048	101.925

Group SE	TARC	TIMP-1
WT NL Single	8.635	9.040
WT HU Single	46.031	113.881
MCAT NL Single	8.386	177.316
MCAT HU Single	7.503	44.906
WT NL Social	18.171	17.511
WT HU Social	18.165	39.366
MCAT NL Social	11.812	22.884
MCAT HU Social	22.816	16.547

Group SD	TARC	TIMP-1
WT NL Single	14.956	15.659
WT HU Single	79.729	197.247
MCAT NL Single	16.773	354.632
MCAT HU Single	15.006	89.811
WT NL Social	44.511	42.894
WT HU Social	44.496	96.427
MCAT NL Social	28.932	56.054
MCAT HU Social	55.887	40.532

Supplementary Table 2. Expression levels of plasma cytokines per group expressed in pg/g tissue. Values shown are means, SD and SE. Sample sizes are: WT NL single (n=6), WT HU single (n=6), MCAT NL single (n=6), MCAT HU single (n=6), WT NL social (n=7), WT HU social (n=5), MCAT NL social (n=6), and MCAT HU social (n=6).

**Group Mean (pg/g)**	**Eotaxin**	**G-CSF**	**GM-CSF**	**IFNy**	**IL-1a**	**IL-1B**	**IL-2**	**IL-3**	**IL-4**
WT NL Single (n=6)	1123.717	325.433	54.613	16.507	377.150	70.361	57.037	12.786	1.854
WT HU Single (n=6)	859.978	396.486	22.292	6.936	223.986	30.808	22.312	5.360	1.504
MCAT NL Single (n=6)	1024.025	240.558	27.895	8.035	239.840	30.682	30.017	6.175	0.875
MCAT HU Single (n=6)	1005.255	198.782	31.147	8.240	270.473	49.030	37.338	6.888	1.493
WT NL Social (n=7)	819.892	203.240	31.310	6.842	303.642	32.180	28.775	5.385	0.747
WT HU Social (n=5)	729.332	415.025	23.327	4.162	207.917	24.263	22.205	3.568	0.750
MCAT NL Social (n=6)	701.253	213.528	14.735	2.560	159.230	18.363	22.362	3.097	0.455
MCAT HU Social (n=6)	949.512	257.903	68.093	22.557	473.767	98.958	76.307	17.972	3.258

**Group SE**	**Eotaxin**	**G-CSF**	**GM-CSF**	**IFNy**	**IL-1a**	**IL-1B**	**IL-2**	**IL-3**	**IL-4**
WT NL Single	77.924	112.269	17.414	7.786	140.474	28.491	22.426	6.099	0.832
WT HU Single	69.235	181.787	17.188	6.696	122.815	26.657	19.208	5.226	0.877
MCAT NL Single	76.133	49.291	17.024	7.150	134.034	26.208	25.936	5.786	0.787
MCAT HU Single	46.860	50.288	17.328	5.211	125.914	28.496	19.567	4.282	0.991
WT NL Social	97.377	34.571	14.133	5.836	136.161	21.143	15.594	4.315	0.591
WT HU Social	114.874	157.694	10.438	3.842	77.012	14.059	9.120	2.815	0.431
MCAT NL Social	52.729	53.461	10.304	2.240	66.406	12.781	13.821	2.965	0.291
MCAT HU Social	50.708	65.293	23.052	11.740	190.677	41.915	33.198	9.117	1.269

**Group SD**	**Eotaxin**	**G-CSF**	**GM-CSF**	**IFNy**	**IL-1a**	**IL-1B**	**IL-2**	**IL-3**	**IL-4**
WT NL Single	206.169	297.036	46.073	20.600	371.659	75.380	59.333	16.137	2.201
WT HU Single	154.815	406.487	38.434	14.973	274.623	59.607	42.949	11.685	1.961
MCAT NL Single	186.487	120.737	41.701	17.514	328.315	64.195	63.529	14.173	1.927
MCAT HU Single	114.784	123.179	42.444	12.765	308.424	69.801	47.929	10.489	2.427
WT NL Social	238.524	84.681	34.619	14.296	333.525	51.789	38.197	10.570	1.448
WT HU Social	281.384	386.271	25.567	9.412	188.640	34.438	22.339	6.896	1.056
MCAT NL Social	129.160	130.952	25.238	5.488	162.662	31.307	33.854	7.262	0.713
MCAT HU Social	124.208	159.935	56.466	28.758	467.062	102.671	81.317	22.333	3.109

Supplementary Table 2 continued

Group Mean (pg/g)	IL-17	IP-10	KC	LIF	LIX	MCP-1	M-CSF	MIG	MIP-1a
WT NL Single (n=6)	5.777	115.147	255.864	13.323	1868.846	113.769	380.299	49.154	222.251
WT HU Single (n=6)	3.084	54.838	117.338	2.478	1575.494	53.038	153.802	39.754	143.842
MCAT NL Single (n=6)	2.612	89.542	146.808	2.688	2173.327	63.912	186.762	41.498	144.898
MCAT HU Single (n=6)	3.802	86.535	132.600	3.573	1165.845	75.077	216.417	31.672	165.092
WT NL Social (n=7)	2.784	80.263	145.210	2.411	935.296	53.329	165.638	36.015	185.200
WT HU Social (n=5)	4.193	86.883	105.773	4.103	1786.215	51.287	151.473	67.803	154.190
MCAT NL Social (n=6)	1.512	97.698	109.845	1.473	693.732	34.608	104.458	31.652	133.153
MCAT HU Social (n=6)	8.840	118.742	231.607	7.948	1809.173	142.548	557.012	46.595	258.348

Group SE	IL-17	IP-10	KC	LIF	LIX	MCP-1	M-CSF	MIG	MIP-1a
WT NL Single	2.307	21.213	61.490	9.155	926.429	36.349	157.771	9.058	46.619
WT HU Single	1.956	18.317	46.033	2.318	1033.124	40.375	131.746	9.182	45.973
MCAT NL Single	2.226	7.875	72.049	2.688	875.750	36.521	163.348	5.649	49.516
MCAT HU Single	2.239	10.596	50.518	2.172	399.022	40.497	123.358	2.979	48.503
WT NL Social	1.674	9.315	39.422	1.606	279.378	25.196	119.196	4.396	38.322
WT HU Social	2.171	13.895	41.823	2.248	619.484	21.691	77.710	25.362	28.174
MCAT NL Social	0.943	10.383	45.441	1.085	234.940	19.145	69.025	2.070	30.838
MCAT HU Social	3.575	19.201	91.254	3.739	963.028	50.852	270.201	6.186	59.843

Group SD	IL-17	IP-10	KC	LIF	LIX	MCP-1	M-CSF	MIG	MIP-1a
WT NL Single	6.105	56.125	162.688	24.222	2451.102	96.172	417.424	23.965	123.342
WT HU Single	4.373	40.959	102.933	5.182	2310.136	90.281	294.594	20.532	102.799
MCAT NL Single	5.451	19.289	176.484	6.585	2145.140	89.457	400.118	13.836	121.288
MCAT HU Single	5.485	25.956	123.744	5.321	977.401	99.196	302.163	7.296	118.807
WT NL Social	4.428	24.645	104.301	4.248	739.164	66.662	291.971	10.768	93.868
WT HU Social	5.317	34.035	102.445	5.507	1517.421	53.131	190.349	62.123	69.012
MCAT NL Social	2.309	25.433	111.308	2.658	575.483	46.897	169.076	5.069	75.537
MCAT HU Social	8.757	47.034	223.526	9.159	2358.928	124.562	661.854	15.153	146.586

Supplementary Table 2
continued

Group Mean (pg/g)	MIP-1B	MIP-2	RANTES	TNFa	VEGF	EPO	6Ckine/Exodus 2	Fractalkine
WT NL Single (n=6)	151.636	692.454	65.407	32.977	3.164	288.863	5894.476	543.724
WT HU Single (n=6)	62.314	339.476	24.820	15.812	1.276	226.270	5143.190	394.784
MCAT NL Single (n=6)	75.852	329.288	37.333	21.885	1.487	234.060	5389.305	256.407
MCAT HU Single (n=6)	87.270	433.132	46.672	25.050	1.573	272.445	4664.130	259.532
WT NL Social (n=7)	92.838	338.147	37.680	18.698	1.448	224.353	8476.576	333.685
WT HU Social (n=5)	77.583	266.612	37.730	17.182	1.148	621.813	5183.263	469.495
MCAT NL Social (n=6)	44.067	252.837	29.538	14.668	1.052	465.122	13105.610	383.120
MCAT HU Social (n=6)	176.610	832.323	67.915	39.852	3.017	428.570	5684.690	359.407

Group SE	MIP-1B	MIP-2	RANTES	TNFa	VEGF	EPO	6Ckine/Exodus 2	Fractalkine
WT NL Single	39.203	213.868	15.956	9.690	1.121	46.079	829.925	209.169
WT HU Single	47.539	228.024	16.069	8.766	0.640	63.486	1765.812	49.351
MCAT NL Single	44.278	229.544	18.942	9.447	0.835	55.429	992.100	17.324
MCAT HU Single	45.709	208.143	17.614	9.759	0.667	32.195	1044.404	16.702
WT NL Social	34.212	202.560	12.687	7.836	0.546	59.261	2564.503	16.042
WT HU Social	26.593	159.480	9.696	5.303	0.386	321.363	1035.023	174.920
MCAT NL Social	29.671	148.845	10.952	4.762	0.564	129.363	7172.224	33.344
MCAT HU Social	57.735	254.951	24.693	14.001	1.236	98.904	1620.982	54.333

Group SD	MIP-1B	MIP-2	RANTES	TNFa	VEGF	EPO	6Ckine/Exodus 2	Fractalkine
WT NL Single	103.721	565.841	42.216	25.638	2.967	121.913	2195.774	553.410
WT HU Single	106.300	509.878	35.932	19.600	1.431	141.958	3948.475	110.351
MCAT NL Single	108.459	562.266	46.398	23.140	2.045	135.772	2430.139	42.435
MCAT HU Single	111.965	509.844	43.146	23.905	1.635	78.862	2558.257	40.911
WT NL Social	83.802	496.168	31.077	19.195	1.337	145.160	5734.403	39.294
WT HU Social	65.139	390.644	23.750	12.991	0.946	787.176	2535.279	428.465
MCAT NL Social	72.678	364.595	26.827	11.666	1.380	316.873	14344.447	81.676
MCAT HU Social	141.421	624.500	60.485	34.295	3.028	242.263	3624.625	133.088

Supplementary Table 2 continued

Group Mean (pg/g)	IFNB-1	IL-11	IL-16	IL-20	MDC	MCP-5	MIP-3a	MIP-3B	TARC
WT NL Single (n=6)	224.380	158.059	1314.753	663.114	362.944	100.099	75.450	247.581	96.803
WT HU Single (n=6)	206.394	75.850	1576.838	802.820	161.230	52.336	57.930	244.046	61.648
MCAT NL Single (n=6)	66.235	15.653	1522.603	377.902	273.268	99.738	49.238	201.163	71.390
MCAT HU Single (n=6)	72.680	18.417	1638.043	634.688	243.643	73.740	45.332	308.085	59.788
WT NL Social (n=7)	100.658	31.433	1978.345	413.628	188.523	105.667	62.918	208.595	52.133
WT HU Social (n=5)	197.490	228.453	2273.075	786.843	165.510	85.440	76.218	197.187	86.895
MCAT NL Social (n=6)	138.332	44.995	1522.627	515.365	151.125	84.212	71.367	354.707	55.503
MCAT HU Social (n=6)	136.100	49.005	1814.538	617.173	169.825	76.827	79.472	281.875	65.360

Group SE	IFNB-1	IL-11	IL-16	IL-20	MDC	MCP-5	MIP-3a	MIP-3B	TARC
WT NL Single	127.625	114.152	290.516	217.067	70.138	23.710	13.290	46.426	23.900
WT HU Single	40.134	23.730	350.728	55.146	22.527	12.658	8.145	35.405	15.287
MCAT NL Single	9.301	4.011	302.486	48.032	28.458	23.105	5.823	12.309	8.365
MCAT HU Single	10.807	5.178	255.468	145.627	37.624	11.945	3.410	70.018	7.288
WT NL Social	18.066	6.915	234.012	57.004	37.173	27.050	11.084	17.679	8.586
WT HU Social	76.404	166.451	668.557	78.394	43.095	15.511	9.345	31.072	14.333
MCAT NL Social	22.631	19.451	259.402	85.505	31.190	7.915	7.147	119.057	3.626
MCAT HU Social	46.021	17.809	201.943	52.380	35.783	8.362	18.895	79.511	7.569

Group SD	IFNB-1	IL-11	IL-16	IL-20	MDC	MCP-5	MIP-3a	MIP-3B	TARC
WT NL Single	337.665	302.017	768.632	574.305	185.567	62.731	35.163	122.833	63.235
WT HU Single	89.742	53.062	784.252	123.310	50.372	28.305	18.214	79.169	34.183
MCAT NL Single	22.783	9.825	740.936	117.653	69.707	56.595	14.262	30.152	20.489
MCAT HU Single	26.470	12.683	625.766	356.713	92.160	29.259	8.353	171.508	17.851
WT NL Social	44.254	16.937	573.210	139.632	91.055	66.258	27.150	43.305	21.031
WT HU Social	187.150	407.721	1637.624	192.026	105.560	37.994	22.890	76.109	35.110
MCAT NL Social	55.435	47.645	635.404	209.444	76.399	19.388	17.506	291.628	8.883
MCAT HU Social	112.729	43.623	494.656	128.304	87.651	20.483	46.283	194.762	18.541

Supplementary Table 2 continued

Group Mean (pg/g)	TIMP-1
WT NL Single (n=6)	2363.110
WT HU Single (n=6)	1415.994
MCAT NL Single (n=6)	1417.617
MCAT HU Single (n=6)	1836.257
WT NL Social (n=7)	2636.078
WT HU Social (n=5)	6015.570
MCAT NL Social (n=6)	3304.292
MCAT HU Social (n=6)	1794.837

Group SE	TIMP-1
WT NL Single	762.909
WT HU Single	308.464
MCAT NL Single	119.452
MCAT HU Single	289.634
WT NL Social	189.253
WT HU Social	3045.488
MCAT NL Social	645.855
MCAT HU Social	285.087

Group SD	TIMP-1
WT NL Single	2018.467
WT HU Single	689.746
MCAT NL Single	292.598
MCAT HU Single	709.456
WT NL Social	463.574
WT HU Social	7459.891
MCAT NL Social	1582.015
MCAT HU Social	698.318

Supplementary Tables 3 to 13

Supplementary Table 3. Two-way ANOVA was performed for main factors isolation, genotype and simulated microgravity. The only statistically significant interaction was isolation x genotype. Group comparisons which are not presented are not statistically significant. The means of the four groups (NL and HU groups combined) in the isolation x genotype analysis are shown as the first four columns of values. The interaction p-values are shown in the next column. The last two columns pertain to the statistically significant Tukey post hoc p-values from the comparison of the four groups shown in the table. Only cytokines that show statistically significant differences across the four groups are shown in the table.

Cytokine	Mean (pg/g)				Interaction p-value	Post hoc p-value	
	WT Single	WT Social	MCAT Single	MCAT Social	Isolation x Genotype	WT social vs WT single	WT single vs MCAT single
**IL-6**	13.5	3.42	2.5	3.84	0.0067	0.0001	0.0001
**INF-γ**	163	77.2	61.8	89.8	0.0007	0.0001	0.0001
**MCP-1**	3150	1230	1310	1480	0.0117	0.0002	0.0002
**IL-9**	363	199	222	241	0.0051	0.0001	0.0011
**IL-13**	692	196	170	206	0.0151	0.0001	0.0001
**LIF**	5.62	2.1	3.34	3.62	0.03	0.0498	0.060
**IL-4**	3.84	1.52	0.48	2.16	0.0009	0.0041	0.0001
**M-CSF**	70.6	38.5	44.5	51.1	0.008	0.0018	0.039
**TNF-α**	12.4	1.07	2.19	2.56	0.03	0.005	0.0031
**MIG**	327	233	240	288	0.0083	0.0072	0.0183
**G-CSF**	8.32	1.97	1.29	3.25	0.0135	0.0056	0.0038

Supplementary Table 4. Two-way ANOVA was performed for main effects genotype and simulated microgravity in hippocampus of single housed animals. Only IL-13 showed statistically significant interaction effects for genotype x simulated microgravity. The Tukey post hoc results for this cytokine are shown below. The means of the four groups in the genotype x simulated microgravity analysis are shown as the first four columns of values. All statistically significant p-values and the group values are presented in the table below. Group comparisons with no statistically significant differences are not shown.

Cytokine	Mean (pg/mg)				Interaction p-value	Post hoc p-value		
	WT NL	WT HU	MCAT NL	MCAT HU	Genotype x Microgravity	WT NL vs WT HU	WT NL vs MCAT NL	WT HU vs MCAT HU
**IL-13**	0.874	0.509	0.133	0.206	0.0086	0.0214	0.0001	0.0406

Supplementary Table 5. The two-way ANOVA performed above (Suppl Table 4) also revealed a genotype effect in 13 cytokines. The WT and MCAT groups were compared by Dunnett's test (genotype effect and Dunnett's p-values are the same). All statistically significant p-values and the group values are presented in the table below. Group comparisons with no statistically significant differences are not shown. All MCAT group values were lower compared to WT.

Cytokine	Mean (pg/g)		Genotype effect p-value (Dunnett's)
	WT	MCAT	WT vs MCAT
**Inf-γ**	154	61	0.0001
**G-CSF**	8.29	1.48	0.0034
**IL-4**	3.27	0.484	0.0001
**IL-6**	12.8	2.49	0.0001
**IL-7**	36	20	0.037
**IL-9**	3445	2217	0.0058
**IL-10**	171	18	0.0047
**IL-13**	698	170	0.0003
**IL-17**	12.6	8.6	0.04
**IP-10**	216	111	0.011
**MCP-1**	2980	1300	0.0037
**M-CSF**	65	46	0.04
**RANTES**	39	25	0.0057

Supplementary Table 6. Two-way ANOVA was performed for main effects genotype and simulated microgravity in hippocampus of social housed animals. Five cytokines showed statistically significant interaction effects for genotype x simulated microgravity. The Tukey post hoc results for these five cytokines are shown below. The means of the four groups in the genotype x simulated microgravity analysis are shown as the first four columns of values. All statistically significant p-values and the group values are presented in the table below. Group comparisons with no statistically significant differences are not shown.

Cytokine	Mean (pg/g)				Interaction p-value	Post hoc p-value
	WT NL	WT HU	MCAT NL	MCAT HU	Genotype x Microgravity	WT NL vs WT HU
**IL-3**	1.44	4.42	2.99	1.91	0.002	0.01
**IL-1B**	176	276	223	205	0.05	0.036
**IL-10**	90.7	132	162	123	0.0132	0.026
**IL-12 (p70)**	66.7	33.7	53.9	43.1	0.0162	0.0367
**IL-17**	9.14	12.1	14.4	12.3	0.043	0.0338

Supplementary Table 7. Two-way ANOVA was performed for main effects genotype and simulated microgravity in plasma of single housed animals. Three cytokines showed statistically significant interaction effects for genotype x microgravity. The Tukey post hoc results for these three cytokines are shown below. The means of the four groups in the genotype x simulated microgravity analysis are shown as the first four columns of values. All statistically significant p-values and the group values are presented in the table below. Group comparisons with no statistically significant differences are not shown.

Cytokine	Mean (pg/ml)				Interaction p-value	Post hoc p-value	
	WT NL	WT HU	MCAT NL	MCAT HU	Genotype x Microgravity	WT NL vs WT HU	WT HU vs MCAT HU
**IL-20**	455	808	378	505	0.045	0.0075	0.0307
**MDC**	363	181	273	244	0.028	0.0412	ns
**INF-β**	98	243	66.2	72.2	0.009	0.0137	0.0023

Supplementary Table 8. Two-way ANOVA of plasma from single housed animals performed above (Suppl Table 7) also revealed a genotype effect in one cytokine. The WT and MCAT groups (regardless of loading state) were compared using a Dunnett's test. All statistically significant p-values and the group values are presented in the table below. Group comparisons with no statistically significant differences are not shown. All MCAT groups values were lower compared to WT.

Cytokine	Mean (pg/ml)		Genotype effect p-value (Dunnett's)
	WT	MCAT	WT vs MCAT
**Fractalkine**	372	258	0.0009

Supplementary Table 9. Two-way ANOVA was performed for main effects genotype and simulated microgravity in plasma of social housed animals. Only one cytokine showed statistically significant interaction effects for genotype x simulated microgravity. The Tukey post hoc results for this cytokine is shown below. The means of the four groups in the genotype x microgravity analysis are shown as the first 4 columns of values. All statistically significant p-values and the group values are presented in the table below. Group comparisons with no statistically significant differences are not shown. Only cytokines that show statistically significant differences across the four groups are shown in the table.

Cytokine	Mean (pg/ml)				Interaction p-value	Post hoc p-value
	WT NL	WT HU	MCAT NL	MCAT HU	Genotype x Microgravity	WT NL vs WT HU
**IL-20**	414	901	449	617	0.0263	0.0059

Supplementary Table 10. Two-way ANOVA was performed for main factors isolation, genotype and simulated microgravity in plasma. No interaction effects were found. However, a housing effect was observed in two cytokines. The means of the single and social housed groups (regardless of loading state and genotype) are shown. The p-values for the main effects are shown.

Cytokine	Mean (pg/ml)		Isolation effect p-value (Dunnett's)
	Single	Social	Social vs Single
**Eotaxin**	1014	800	0.0007
**MDC**	269	169	0.004

Supplementary Table 11. Nonparametric Wilcoxon (data variances not equal) all pairs was applied to plasma corticosterone values. The p-values for each comparison are shown in the last column. *Statistically significant at p<0.05.

**Group comparisons**		**p-value**
WT HU Social	MCAT NL Single	**0.0003***
MCAT HU Social	MCAT NL Single	**0.0003***
WT HU Social	WT NL Single	**0.0008***
MCAT HU Social	WT NL Single	**0.0008***
MCAT NL Social	MCAT NL Single	**0.0009***
MCAT HU Social	WT NL Social	**0.0029***
MCAT NL Social	WT NL Single	**0.0028***
MCAT NL Social	WT NL Social	**0.0136***
WT HU Social	WT NL Social	**0.0283***
MCAT HU Social	MCAT HU Single	**0.0372***
WT NL Social	WT NL Single	**0.0406***
MCAT HU Single	WT NL Single	**0.0428***
WT HU Single	WT NL Single	**0.0481***
MCAT NL Single	WT HU Single	**0.0481***
MCAT HU Single	MCAT NL Single	0.0728
MCAT NL Social	MCAT HU Single	0.0933
WT NL Social	MCAT NL Single	0.1149
MCAT NL Single	WT NL Single	0.1563
MCAT HU Social	WT HU Social	0.3078
WT HU Social	MCAT HU Single	0.3179
MCAT NL Social	WT HU Social	0.6789
MCAT HU Social	MCAT NL Social	0.9013
MCAT NL Social	WT HU Single	0.8262
MCAT HU Social	WT HU Single	0.7332
WT HU Social	WT HU Single	0.5697
WT NL Social	MCAT HU Single	0.4175
MCAT HU Single	WT HU Single	0.2556
WT NL Social	WT HU Single	0.1643

Supplementary Table 12. Two-way ANOVA of plasma 4-HNE levels for main factors isolation, genotype and simulated microgravity was performed. A housing effect was observed. The means of the single and social housed groups (regardless of loading state and genotype) are shown. The p-values for the main effect housing are shown.

	**Mean (%)**		**Housing effect p-value (Dunnett's)**
	**Social**	**Single**	**Social vs Single**
**4-HNE**	15.5	21	0.0432

Supplementary Table 13. In the two-way ANOVA of plasma 4-HNE levels for main factors isolation, genotype and simulated microgravity performed above (Table 12), a statistically significant interaction effect for genotype x housing was found. The Tukey post hoc is shown below in the last column. The means of the four groups (NL and HU groups combined) in the genotype x housing analysis are shown as the first four columns of values. All statistically significant p-values and the group values are presented in the table below. Group comparisons with no statistically significant differences are not shown.

	**Mean (%)**				**Interaction p-value**	**Post hoc p-value**
	**WT Social**	**WT Single**	**MCAT Social**	**MCAT Single**	**Genotype x Housing**	**WT Single vs WT Social**
**4-HNE**	11	23	19	18	0.0148	0.0147
