# Supplementary material for: Overexpression of catalase in mitochondria mitigates changes in hippocampal cytokine expression following simulated microgravity and isolation
Source: NPJ Microgravity. 2021 Jul 6;7:24. doi: 10.1038/s41526-021-00152-w (PMC8260663; doi:10.1038/s41526-021-00152-w)
Supplement: Supplementary file 2 — Reporting Summary [file 41526_2021_152_MOESM2_ESM.pdf]

nature research

Corresponding author(s): Candice Tahimic

Last updated by author(s): May 30, 2021

Reporting Summary

Nature Research wishes to improve the reproducibility of the work that we publish. This form provides structure for consistency and transparency in reporting. For further information on Nature Research policies, see our Editorial Policies and the Editorial Policy Checklist.

Statistics

For all statistical analyses, confirm that the following items are present in the figure legend, table legend, main text, or Methods section.

n/a	Confirmed	
☐	☒	The exact sample size (*n*) for each experimental group/condition, given as a discrete number and unit of measurement
☐	☒	A statement on whether measurements were taken from distinct samples or whether the same sample was measured repeatedly
☐	☒	The statistical test(s) used AND whether they are one- or two-sided<br>*Only common tests should be described solely by name; describe more complex techniques in the Methods section.*
☐	☒	A description of all covariates tested
☐	☒	A description of any assumptions or corrections, such as tests of normality and adjustment for multiple comparisons
☐	☒	A full description of the statistical parameters including central tendency (e.g. means) or other basic estimates (e.g. regression coefficient) AND variation (e.g. standard deviation) or associated estimates of uncertainty (e.g. confidence intervals)
☐	☒	For null hypothesis testing, the test statistic (e.g. *F*, *t*, *r*) with confidence intervals, effect sizes, degrees of freedom and *P* value noted<br>*Give P values as exact values whenever suitable.*
☒	☐	For Bayesian analysis, information on the choice of priors and Markov chain Monte Carlo settings
☒	☐	For hierarchical and complex designs, identification of the appropriate level for tests and full reporting of outcomes
☒	☐	Estimates of effect sizes (e.g. Cohen's *d*, Pearson's *r*), indicating how they were calculated

Our web collection on statistics for biologists contains articles on many of the points above.

Software and code

Policy information about availability of computer code

Data collection	Not applicable
Data analysis	Not applicable

For manuscripts utilizing custom algorithms or software that are central to the research but not yet described in published literature, software must be made available to editors and reviewers. We strongly encourage code deposition in a community repository (e.g. GitHub). See the Nature Research guidelines for submitting code & software for further information.

Data

Policy information about availability of data

All manuscripts must include a data availability statement. This statement should provide the following information, where applicable:

- Accession codes, unique identifiers, or web links for publicly available datasets
- A list of figures that have associated raw data
- A description of any restrictions on data availability

All data generated or analyzed during this study are included in this published article and its supplementary information files.

Field-specific reporting

Please select the one below that is the best fit for your research. If you are not sure, read the appropriate sections before making your selection.

☒ Life sciences ☐ Behavioural & social sciences ☐ Ecological, evolutionary & environmental sciences

For a reference copy of the document with all sections, see nature.com/documents/nr-reporting-summary-flat.pdf

Life sciences study design

All studies must disclose on these points even when the disclosure is negative.

Sample size	We used sample sizes that we found to be optimal based on previous studies.
Data exclusions	We set a criteria before viewing the data that data points that were 2.5 SDs from the mean will be eliminated. However, we rarely encountered such outliers in this study.
Replication	We used a suitable sample size consisting of unique animal subjects within a group. We have not conducted a repeat of the experiment using the same study design. However, we have conducted an unrelated study involving a separate set of wild type normally loaded and wild type hindlimb unloaded animals of similar age, sex and strain (PMID: 33921854). We found that the cytokine and corticosterone results generally were consistent between the two studies.
Randomization	We assigned animals to generate similar starting mean body weights per group prior to hindlimb unloading.
Blinding	All operators of the various experimental assays were blinded to the identities of the samples they were processing/analyzing.

Reporting for specific materials, systems and methods

We require information from authors about some types of materials, experimental systems and methods used in many studies. Here, indicate whether each material, system or method listed is relevant to your study. If you are not sure if a list item applies to your research, read the appropriate section before selecting a response.

Materials & experimental systems

n/a	Involved in the study	
☐	☒	Antibodies
☐	☐	Eukaryotic cell lines
☒	☐	Palaeontology and archaeology
☐	☒	Animals and other organisms
☐	☐	Human research participants
☒	☐	Clinical data
☒	☐	Dual use research of concern

Methods

n/a	Involved in the study	
☒	☐	ChIP-seq
☒	☐	Flow cytometry
☒	☐	MRI-based neuroimaging

Antibodies

Antibodies used	Corticosterone ELISA assay kit (Abcam; cat# ab108821); OxiSelect HNE Adduct Competitive ELISA (Cell Biolabs, Cat# STA-838); Eve Technologies' cytokine assay uses proprietary methods for protein level detection
Validation	Corticosterone and 4-HNE standards were provided by the manufacturers of the respective assays which the first author run together with the samples. Negative controls (PBS only) were also included by the first author when performing both assays. Eve Technologies provides a standard curve for each of the cytokines measured in the panel. The cytokine absorbance results also were compared to the values obtained from a negative control (lysis buffer only) to determine threshold for detection of cytokines.

Eukaryotic cell lines

Policy information about cell lines

Cell line source(s)	Not applicable
Authentication	Not applicable
Mycoplasma contamination	Not applicable
Commonly misidentified lines (See ICLAC register)	Not applicable

Animals and other organisms

Policy information about studies involving animals; ARRIVE guidelines recommended for reporting animal research

Laboratory animals	Male MCAT mice, B6.Cg-Tg (CAG-OTC/CAT) 4033Prab/J and female C57BL/6NJ mice (from Jackson Laboratory; stocks 016197 and 005304 respectively) were crossed to generate the experimental animals for this study
Wild animals	Not applicable
Field-collected samples	Not applicable
Ethics oversight	NASA Ames Research Center IACUC; Animal protocol numbers NAS-16-006 and NAS-16-007

Note that full information on the approval of the study protocol must also be provided in the manuscript.

Human research participants

Policy information about studies involving human research participants

Population characteristics	Not applicable
Recruitment	Not applicable
Ethics oversight	Not applicable

Note that full information on the approval of the study protocol must also be provided in the manuscript.
